# Sepsis due to *Actinomyces odontolyticus* as a Rare Complication of Neobladder

**DOI:** 10.1155/2021/6699046

**Published:** 2021-03-02

**Authors:** Bashar Khiatah, Kalpan Shah, Anna Belikova, Musab Saeed

**Affiliations:** ^1^Department of Internal Medicine, Community Memorial Hospital, 147 N Brent St, Ventura, CA 93003, USA; ^2^Department of Internal Medicine, Community Memorial Hospital, Ventura, CA 93003, USA; ^3^Department of Infectious Disease, Community Memorial Hospital, Ventura, CA 93003, USA

## Abstract

Sepsis due to *Actinomyces odontolyticus* (*A. odontolyticus*) is an extremely rare condition that has been reported only a handful of times. In this report, we showcase an 83-year-old male who had a complication of neobladder procedure and developed sepsis due to *A. odontolyticus* that was successfully treated with a prolonged course of doxycycline.

## 1. Introduction

*Actinomyces* species are filamentous Gram-positive bacilli, a main component of the oropharynx, gastrointestinal tract, and urogenital tract flora. Colonization is omnipresent in healthy individuals, and suppurative infections usually tend to happen in individuals who are immunosuppressed with poor oral hygiene and after disruption of local tissue. More than 30 species of *Actinomyces* have been identified. The most common clinical form of actinomycosis is cervicofacial invasive infection [1] *A. odontolyticus* is one of these species that has been first found in the dentine and later on reported to form rust-brown or red-colored colonies in laboratory studies where it is further characterized by facultative capnophilic, nonsporulating, nonacid fast, nonmotile, and irregularly staining bacteria [2] *A. odontolyticus*, besides *A. meyeri* and *A. israelii*, has been reported to have an extremely rare kind of *Actinomyces* that is hematogenously disseminated [3]. Few cases so far have been reported of bacteremia with *A. odontolyticus* [4, 5]. In this report, we showcase an 83-year-old male who had a complication of a cystoprostatectomy with the creation of neobladder and developed acute bacteremia with *A. odontolyticus*.

## 2. Clinical Case

An 83-year-old male presents to the Emergency Room (ER) for abdominal pain, fever, and rigors for one day. His past medical history is notable for elevated prostate-specific antigen (PSA) on outpatient labs, with subsequent magnetic resonance imaging (MRI) of prostate revealing 12-millimeter (mm) lesion at the left lateral transitional zone and enlarged prostate which was assessed with the following cystoscopy that revealed bladder cancer throughout the bladder wall, which for the patient underwent transurethral resection of bladder tumor. Final pathology revealed high-grade urothelial carcinoma with lamina propria and deep muscularis propria invasion. The patient underwent uncomplicated cystoprostatectomy 2 weeks before presenting to our ER, with the subsequent creation of an ileal neobladder and placement of a Foley catheter and double-J ureteral stents.

His current symptoms began one day before presentation after he stepped on his Foley catheter, causing it to dislodge into the urethra. He was febrile on admission (temperature 101.1 F), and his clinical exam was remarkable for a soft abdomen without peritoneal signs (no voluntary guarding and no rebound tenderness). Initial computed tomography (CT) abdomen/pelvis without contrast showed an inflated Foley catheter balloon in the urethra with no signs of soft tissue infection found that would suggest a soft tissue. Attempts to reposition the catheter by ER nurse were unsuccessful, and repeated CT abdomen/pelvis continued to show the Foley catheter in the urethra. The urology team was consulted, who deflated the balloon and appropriately placed the catheter in the bladder with resultant spontaneous voiding of bloody urine. A follow-up cystogram showed a negligible amount of urine at the level of anastomosis from a traumatized balloon, with an intact bladder and appropriate position of the catheter in the bladder. His initial admission laboratory tests are listed in Table 1. The patient was admitted for severe urosepsis (fever, elevated white blood cell count in immunocompromised state, lactic acidosis, and leukocyte esterase and nitrites seen on urinalysis), had blood cultures and urine culture collected, and was empirically started on intravenous (IV) ciprofloxacin. His blood cultures subsequently came back positive for *A. odontolyticus* in 2 out of 2 sets of culture bottles, and the infectious disease team was consulted. His ciprofloxacin was switched to ceftriaxone and doxycycline due to remote history of allergy. The patient remained asymptomatic without fever and chills throughout the rest of the hospitalization. His repeated blood cultures and urine cultures did not show any growth. His ureteral stents and Foley catheter were removed during the hospitalization, and he was able to void appropriately without any catheter. He was discharged with a PICC (peripherally inserted central catheter) line in place, to continue ceftriaxone 2 g intravenously every 24 hours and doxycycline 100 mg orally twice daily for 4 weeks. He was also given a prescription to obtain weekly complete blood counts (CBCs) and chemistry panel (CMP) and was scheduled for an outpatient follow-up with our infectious disease clinic and with our urology clinic.

On follow-up appointment, the patient recovered well and completed the full course of IV and oral antibiotics without any complications. He was placed on oral doxycycline for an extended period that is intended to be for at least 6 months.

## 3. Discussion

While multiple forms of *A. odontolyticus* culture-positive invasive infections have been reported, the incidence of *A. odontolyticus* bacteremia remains extremely rare. Immunosuppression is essential or having a significant risk factor for infection, including poor oral hygiene, intravenous drug use, and previous mucosal manipulation to develop bacteremia with *A. odontolyticus* [4–6]. Gram staining and pathology of infected tissue is usually more sensitive than the culture since it is often difficult to identify in the lab and can be culture-negative and might remain sterile in more than 50% of cases, but when combined with immunofluorescence technique, they provide a highly specific diagnosis [7]. Beta-lactams and especially penicillin G or amoxicillin are treatments of choice for *Actinomyces* spp. Hence, they are extremely susceptible to this spectrum of antibiotics. In case of allergies to these antibiotics, doxycycline, clindamycin, ceftriaxone, and macrolides have been successfully used to treat actinomycosis [1]. The duration recommendation of IV antibiotic therapy is 2 to 6 weeks followed by oral antibiotics for 6 to 12 months in certain individuals which are warranted for complicated and invasive cases, immunocompromised patients, and patients with HIV infection. While shorter courses have been successful in treating actinomycosis, this extended therapy is recommended due to the infection tends to recur [8–10]. Also, another argument supporting prolonged antibiotic treatment is the tendency of *Actinomyces* species to form fistulas that have been reported in many case reports [11, 12]. In our case, the patient is immunocompromised and had an invasive surgery (neobladder), which was probably colonized with *A. odontolyticus*, which has introduced *A. odontolyticus* to his bloodstream causing sepsis. The current literature provides guideline therapy for a severe localized infection rather than sepsis due to *A. odontolyticus*. Thus, given the patient's case, we have determined to proceed with 4 weeks of oral and IV antibiotics (doxycycline and ceftriaxone) followed by 6 months of oral antibiotics (doxycycline) with periodic monitoring of his progress. It might have been worth culturing the patient's urine before the neobladder procedure to detect and prevent this infection, especially considering a subsequent immunosuppressant therapy. Although given the incidence rate, it might not be feasible to culture every single patient undergoing a bladder procedure which might raise an already high medical expense in the US. More research is required to maybe develop a sensitive, rapid, and cheap test to detect *A. odontolyticus* which will help to prevent infection and force early specific treatment.

In conclusion, a high suspicion for *A. odontolyticus* is a cause of sepsis in a patient with high-risk factors. *A. odontolyticus* could cause an endogenous infection arising from the colonized oral (most common) or urogenital (rare) mucosal membranes when an individual becomes immunocompromised or has a surgical intervention or injury to the mucosal membrane serving as an entrance to the bloodstream. Extended treatment is recommended in most cases given recurrence rate and tendency to cause a fistula especially in immunocompromised patients.

## Figures and Tables

**Table 1:** Laboratory studies.

Lab test	Value	Units
White blood count	11.9	K/uL
Hemoglobin	9.3	G/DL
Hematocrit	28.2	%
Platelets	298	K/ul
Sodium	134	MEQ/L
Potassium	4.4	MEQ/L
Chloride	109	MEQ/L
Carbon dioxide	19	MEQ/L
Blood urea nitrogen	24	MG/DL
Creatinine	0.98	MG/DL
Troponin	<0.03	NG/ML
Sed rate	33	MM/HR
Lactic acid, venous	2.3	MMOL/L
C-reactive protein	5.5	MG/DL

## Data Availability

The data used to support the findings of this study are included within the main manuscript.
